# *Lactobacillus rhamnosus* GG: An Overview to Explore the Rationale of Its Use in Cancer

**DOI:** 10.3389/fphar.2017.00603

**Published:** 2017-09-01

**Authors:** Giuseppe L. Banna, Francesco Torino, Francesco Marletta, Maria Santagati, Rossella Salemi, Elisa Cannarozzo, Luca Falzone, Francesco Ferraù, Massimo Libra

**Affiliations:** ^1^Division of Medical Oncology, Cannizzaro Hospital Catania, Italy; ^2^Department of Systems Medicine, Chair of Medical Oncology, Tor Vergata University of Rome Rome, Italy; ^3^Division of Radiotherapy, Cannizzaro Hospital Catania, Italy; ^4^Department of Biomedical and Biotechnological Sciences, Section of Microbiology, University of Catania Catania, Italy; ^5^Department of Biomedical and Biotechnological Sciences, Laboratory of Translational Oncology and Functional Genomics, Section of General and Clinical Pathology and Oncology, University of Catania Catania, Italy; ^6^Division of Medical Oncology, San Vincenzo Hospital Taormina, Italy

**Keywords:** *Lactobacillus rhamnosus* GG, probiotics, diarrhea, cancer, chemotherapy, immunotherapy, radiotherapy

## Abstract

Cancer is the second leading cause of death in the western world. In the era of precision medicine, a significant number of cancer patients can be cured with several anti-cancer therapeutic regimens. However, therapy failure may be caused by treatment side effects, such as diarrhea, especially occurring in patients with gastrointestinal or pelvic malignancies. In particular, diarrhea is one of the most frequent gastrointestinal toxicity during cancer treatment and it can result from nearly bot chemo- and radio-therapeutic strategies currently used. Diarrhea has a serious impact on patients’ quality of life and treatment dosing and schedule modification due to its severity can negatively influence treatment outcomes. In this context, probiotics may play an interesting role in several human diseases with an inflammatory bowel involvement and, among these, *Lactobacillus rhamnosus* GG (LGG) is one of the most characterized and utilized. In particular, LGG is able to reverse intestinal dysbiosis and moderate diarrhea. Moreover, preclinical studies have documented its effects in reducing chronic inflammation associated with cancer development. This review summarizes the preclinical results of LGG on cancer cells proliferation and tumor invasion as well as the potential role of LGG use in cancer patients for the prevention and management of diarrhea associated with cancer treatment. Overall, these encouraging data support further investigation on the use of LGG in stratified patients undergoing specific therapeutic protocols, including chemotherapy and pelvic radiotherapy, in order to reduce the development of severe diarrhea and thus improve the adherence to the therapy and patients’ quality of life.

## Introduction

Cancer remains the second leading cause of death in the western world (Siegel et al., 2017). Currently, a significant fraction of cancer patients can benefit from several types of therapeutic strategies. However, toxicities associated to chemotherapy and radiotherapy can negatively impact treatments responses by altering treatment dosing and schedules as well as affecting patient’s quality of life (Banna et al., 2010). Among the gastrointestinal adverse events (AEs) diarrhea is a frequent and potentially fatal toxicity associated with anti-cancer treatment. (Gibson and Stringer, 2009) In fact, diarrhea of any grade occurs in 30–87% of patients receiving chemotherapy and in 20–49% of patients receiving pelvic radiotherapy. The incidence of severe and life-threatening (grade 3/4) diarrhea ranges from 20 to 40% in patients receiving combined chemoradiotherapy (Benson et al., 2004).

In this context, an important role may be played by probiotics, which include lactic acid bacteria (LAB). Probiotics represent the most common supplement therapy used during intestinal dysbiosis. In 2002, the Joint Food and Agriculture Organization of the United Nations/World Health Organization (FAO/WHO) working group defined probiotics as “live microorganisms, which when consumed in adequate amounts, confer a health effect on the host” (FAO/WHO, 2002). Accordingly, the best well-known benefit of probiotics is the restoration of the normal microbiota during the antibiotic therapy (Guandalini, 2011). Moreover, probiotics may offer several prophylactic functions such as improved microbial balance and immuno-enhancement of the gastrointestinal tract (GIT) (Gill et al., 2000; Ashraf and Shah, 2014).

Gut colonization is fundamental for the complete tropism of the small bowel and the colorectal tract, to achieve full maturation of both local and systemic immune defenses, to protect against the breaching of opportunistic infections through the epithelial barrier, as well as to hone the balance between immune response and tolerance toward non-pathogenic agents (Sekirov et al., 2010).

**FIGURE 1:**
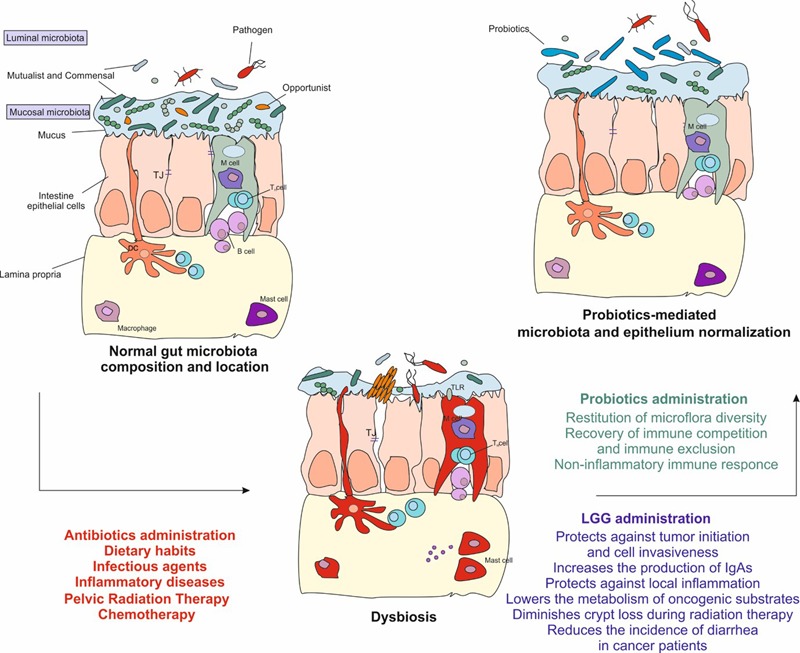
The gut microbiota is fundamental to maintain the intestinal homeostasis. Infectious agents, unhealthy dietary habits, antibiotics, radiation, and chemo or immunotherapy cause the depletion of resident microorganisms. This alteration gives the chance to the transient microbiota, which includes pathogen and opportunistic microorganisms, to breach through the epithelium resulting in a dysbiosis state displayed by abdominal pain, discomfort, bloating, and diarrhea. Dysbiosis eventually leads to the activation of the inflammatory response, to epigenetic modifications and to tissue damage. Administration of probiotics helps to restore the depletion of the gut microbiota and reduces the inflammation. In particular, *Lactobacillus rhamnosus* GG may be helpful in those patients undergoing anti-cancer treatments. It protects against tumor onset and invasiveness, lowers the metabolism of oncogenic substrates, increases the production of IgAs, protects against local inflammation, diminishes crypt loss and apoptosis during radiation therapy. Finally, it has shown to be helpful in reducing diarrhea severity and incidence in cancer patients. TJ, tight junction; DC, dendritic cell; LGG, *Lactobacillus rhamnosus* GG.

Although each individual have a distinctive microbiota (The Human Microbiome Project Consortium, 2012), the gut bacterial population is mainly addressable to Firmicutes and Bacteroidetes (Ley et al., 2006) and it can greatly differ in composition and abundance across the intestinal tract, especially comparing the small intestine to the colon (Zoetendal et al., 2008). Unfortunately, changes in each unique gut microbiota community may induce the condition named dysbiosis as consequence of balance loss among commensal, mutualist, and opportunist/pathobiont microorganisms (**Figure 1**). Commensals and mutualists reside in the host gut by, respectively, showing no known effect and establishing a positive relationship for both the parts. Opportunists and pathogens, in adequate amount or under specific conditions may express their virulence and establish an inflammatory response (Kamada et al., 2013; DeGruttola et al., 2016; Lin and Zhang, 2017). Major features of the onset of dysbiosis are frequently displayed by: reduced number of beneficial microorganisms, increased density of opportunistic colonies and loss of heterogeneity in microbial composition. (DeGruttola et al., 2016). Several factors may trigger changes in each unique composition, these have been related to environmental factors, including diet, resulting in epigenetic changes (Sekirov et al., 2010; Hullar and Fu, 2014; Krautkramer et al., 2016). Unfortunately, dysbiosis is commonly associated with gut diseases characterized by symptoms like nausea, abdominal pain, discomfort, bloating, and diarrhea (Pace et al., 2015; DeGruttola et al., 2016; Lin and Zhang, 2017).

Diarrhea is one of the most common adverse event described during the course of cancer treatments (Mego et al., 2015; McQuade et al., 2016). Depending on its severity it has a serious impact on patients quality of life, on the adherence to treatment and when not properly managed con lead to life-threatening clinical conditions. (Benson et al., 2004; Davis and Milner, 2009; Pace et al., 2015). Several mechanisms are responsible for anti-cancer treatments associated diarrhea. Radiation therapy is a backbone in the treatment of both primary and recurrent pelvic and gastrointestinal malignancies. Despite the advantage of radiotherapy in these settings are widely accepted the damage of normal tissues located within the radiation therapy field can be severe. Abdominal and pelvic irradiation may, at first, trigger extensive crypt loss and direct apoptosis, especially in the region of quiescent stem cells (Ciorba et al., 2012). Gradually, radiation insults lead to the accumulation of reactive oxygen species (ROS), microvascular sclerosis, and ischemia of the tissue and loss of stem cells (Denham and Hauer-Jensen, 2002; Hatoum et al., 2006; Andreyev et al., 2012; Stansborough et al., 2017). The reduced number of cells and crypts, together with microvascular damage are inevitably bound to the impaired functionality of the GIT, especially reducing the absorptive area and leading to the development of diarrhea. These initial alteration amounts to an acute enteritis that can gradually evolve into a chronic atrophy of the intestinal tract also months after treatment is terminated (Berthrong and Fajardo, 1981). The chronical absorption impairment of fats, carbohydrates, protein, bile salts, and electrolytes can lead to malabsorption and diarrhea as well as bacterial overgrowth and ultimately dysbiosis (Wedlake et al., 2008).

Chemotherapy-associated gastrointestinal toxicity is common in cancer patients and diarrhea is the most frequent clinical manifestation. Diarrhea is a known dose-limiting toxicity for cytotoxic agents like fluoropyrimidines (particularly fluorouracile and capecitabine) and irinotecan especially when these are used in association (Lee et al., 2014). Moreover, several targeted therapies, like tyrosine kinase inhibitors and monoclonal antibodies, are associated with diarrhea and more recently immune checkpoint inhibitors have shown to be also responsible for this adverse event (Cramer and Bresalier, 2017).

The rapidly dividing enterocytes are critically affected by common cytotoxic agents which increase apoptosis of epithelial cells in the intestinal mucosa followed by morphometrical changes and finally resulting in the hypoplastic atrophy of small intestine mucosa (Keefe et al., 2000).

These alterations of small bowel lining results in a combination of secretory and osmotic diarrhea (Parnes et al., 1994; Gibson and Stringer, 2009). Moreover, irinotecan and fluoropyrimidines undergo enzymatic modification in liver and gut, therefore gene polymorphisms can greatly affect the availability of the active compounds affecting the toxic effects of these drugs (Stein et al., 2010). Lastly, irinotecan is able to alter the gut microbiota favoring the proliferation of different genera of bacteria. The increased presence of bacterial β-glucuronidase determines increased level of the active metabolite of irinotecan causing significant damage and diarrhea (Stringer et al., 2007).

Up to 50% of patients treated with tyrosine kinase inhibitors develop diarrhea and this is sustained by several mechanisms not always completely elucidated (Keefe and Anthony, 2008; Hirsh et al., 2014). In the case of epidermal growth factor receptor (EGFR) TKIs mediated diarrhea, the inhibition of the EGFR pathway in the intestinal epithelium increases chloride secretion in the intestinal lumen mediated by an increase of intracellular cAMP (Uribe et al., 1996; Kleizen et al., 2000). Therefore, following the electric potential, sodium ions are pulled into the lumen along with water and this abnormal amount of electrolytes impair the physiological absorption of water causing secretory diarrhea.

Immune checkpoint inhibitors directed against CTLA-4 and PD-1 or PD-L1 molecules are also associated with diarrhea. These drugs work by unleashing the immune response to tumor antigens but this can results in decreased tolerance to self antigens leading to inflammatory infiltration of normal tissues including the intestinal tract (Arriola et al., 2016). Clinically, immune checkpoints-induced colitis can range from mild diarrhea to severe colitis with bowel perforation.

An appropriate diet and probiotics administration can prevent and cure dysbiosis, restoring the correct equilibrium of gut microbiota (Delia et al., 2007; Osterlund et al., 2007; Spiller, 2008; Fuccio et al., 2009; Demers et al., 2014).

It was hypothesized that altered gut microbiota promotes and enhances cancer development, especially that of the intestinal tract (Tjalsma et al., 2012). The mechanisms, supporting this hypothesis, are described in several *in vitro* and *in vivo* studies (Tjalsma et al., 2012). Such mechanisms include the production of toxic metabolites that can lead to a chronic inflammatory condition, as well as the biosynthesis of genotoxic compounds interfering with cell cycle control or causing a direct DNA damage through the metabolism of dietary heterocyclic amines (Candela et al., 2011). Of note, chronic inflammation is associated with tumor development. Indeed, the nuclear transcription factor NF-κB, which regulate several pro-inflammatory cytokines, is overexpressed in several cancer types (Mantovani, 2010).

Recently, preclinical and clinical evidences have linked the gut microbiota composition to specific response and toxicity patterns to immune checkpoint inhibitors treatments (Sivan et al., 2015; Vetizou et al., 2015; Chaput et al., 2017; Derosa et al., 2017). In particular, in a melanoma mouse model administration of commensal probiotics has shown to improve tumor control alone and in association with PD-L1 blockade; on the other hand tumors in antibiotic-treated or germ-free mice did not respond to CTLA-4 blockade (Sivan et al., 2015; Vetizou et al., 2015). These evidences have been related to an increased tumor-specific T-cell intervention and intratumoral CD8+ T-cell recruitment in mice with favorable commensal microbiota. Moreover, patients with metastatic renal cell carcinoma who received anti-PD-1 or PD-L1 monotherapy, or the combination of anti-PD-1 plus anti-CTLA-4 inhibitors, and underwent antibiotic therapy the month ahead the immunotherapy treatment, experience lower response rates to immune checkpoint inhibitors (Derosa et al., 2017). In melanoma patients specific gut microbiota patterns have been recently linked to improved response rate to ipilimumab (CTLA-4 inhibitor) but also to an increased occurrence of ipilimumab-induced colitis (Chaput et al., 2017). Taken together these findings support the hypothesis that preventive probiotics treatments may modulate cancer immunotherapy efficacy as well as the impact of specific toxicities and can be associated with the improvement of response rate in cancer patients.

*Lactobacillus* strains are LAB that are widespread in the environment and present in several surface, such as soil, water, and plants. Furthermore, *Lactobacillus* spp. are indigenous commensal inhabitants of the oral cavity, the GIT, and the female genital tract (Coudeyras et al., 2008).

To the best of our knowledge, *Lactobacillus rhamnosus* GG (LGG) (ATCC 53103) is one of the first probiotic studied in cancer and currently used in experimental designs (Goldin et al., 1996). The nomenclature of the species derives from the ability of LGG to metabolize and ferment rhamnose, a biochemical characteristic that is used to identify this species of *Lactobacillus*. To date, *Lactobacillus* spp. are identificated through several methods such as species-specific PCR, PFGE profiling of *L. rhamnosus* GG, bacteriophage typing, proteome analysis using 2D-DIGE and culture methods (i.e., using MRS growth agar medium) (Salminen et al., 2002, 2004, 2006; Koskenniemi et al., 2009; Villion and Moineau, 2009).

Some *L. rhamnosus* strains have beneficial effects on the organism as to be considered probiotics. In particular, LGG is able to withstand gastric acidity and bile salts effectively adhering to the gastrointestinal mucosa. The ability to resist gastric acidity and bile salts is a consequence of the ability of the bacterium to produce anti-stress proteins that give it greater survival capacity in intestinal transit after oral intake (Conway et al., 1987; Goldin et al., 1992). Adherence to the intestinal wall is also favored by the presence on the bacterial wall of exopolysaccharides rich in galactose residues and the presence of specific adhesive pili (Kankainen et al., 2009; Lebeer et al., 2009).

The effect on the immune system is explained by the stimulation and production of different cytokines such as TNF-α, IL-1β, IL-6, IL-10, IL-12, IFN-γ and a particular protein, p40, secreted by *L. rhamnosus* GG cells that can reduce the inflammatory state and apoptosis of intestinal epithelial cells (Claes et al., 2012). Other apoptotic inhibition mechanisms are related to the modulation of cyclooxygenase-2 (COX-2), as will be described later in the manuscript (Korhonen et al., 2004; Ciorba et al., 2012).

Therefore, LGG is well characterized and it is known to have several anti-inflammatory effects (Khailova et al., 2016). Accordingly, treatment with LGG in animal models may reduce the risk of colon cancer through the modulation of the gut microbiota and the downregulation of pro-inflammatory molecules (Gamallat et al., 2016). LGG was first isolated in the late ‘80 from fecal sample of healthy adult (Gorbach, 1996) and subsequently its whole-genome sequence has been well characterized and registered (Morita et al., 2009). In particular, through the comparison of the genome sequence of LGG and other *Lactobacillus* spp., Kankainen et al. (2009) identified LGG-specific islands containing genes coding for three secreted LPXTG-like pilins (spaCBA) and a pilin-dedicated sortase notoriously involved in the mechanisms of adhesion to the intestinal mucosa (Kankainen et al., 2009). It matches the selection criteria for probiotics, including high adhesion *in vitro*, survival trough GIT (gastric acid, bile) and tendency to form colonies with a good persistence in the gut (Gorbach, 1996).

This review summarizes the most relevant preclinical studies describing the effects of LGG on cancer cells proliferation and tumor invasion. Furthermore, its safety in the clinical setting is here described. **Table 1** summarizes the most important studies describing the role of LGG in cancer. Although further clinical studies are needed to better confirm the role of LGG in cancer, we can argue that LGG may be effective in preventing diarrhea during anti-cancer treatments and in increasing the patient compliance to therapy.

**Table 1:** Effects of *Lactobacillus rhamnosus* GG (LGG) in cancer-related intestinal disorders.

Disease	Study model		Effect of LGG	Notes	Reference
	*In vitro*	*In vivo*			
Gastric carcinoma					
	HGC-27		Reduction of the polyamine profile		Linsalata et al., 2010
	AGS		Immunomodulation of IL-8 *H. pylori*dependent		Rokka et al., 2008
Colon cancer					
	Caco-2, HT29, SW480		Pro-apoptotic agent in combination with vitamin K		Orlando et al., 2016
	Caco-2		Immunomodulation of IL-8 flagellin-induced		Lopez et al., 2008
		Male Fischer-344 rats	Antiproliferative	DMH-induced	Goldin et al., 1996
		Sprague Dawley rats	Inhibition of angiogenesis and inflammation	DMH-induced	Gamallat et al., 2016
Metastatic CRC					
	HCT-116		Inhibition of invasiveness		Escamilla et al., 2012
Bladder cancer					
		Female C57BL/6 mice	Tumor regression	Orthopic implantation of MB49	Seow et al., 2010
Intestinal crypt loss					
		C57BL/6 mice	Decrease in epithelial apoptosis	Radiation-induced	Ciorba et al., 2012
Inflammation					
		C57BL/6 mice	Activation of mucosal immune response		Wang et al., 2017
RIGT and CIGT					
		Human	15% lower incidence of diarrhea		Osterlund et al., 2007

*AGS, gastric adenocarcinoma; *H. pylori, Helicobacter pylori*; DMH, Dimethylhydrazine; CRC, colorectal cancer; RIGT, radiation-induced gut toxicity; CIGT, chemo-induced gut toxicity.*

## LGG in Cancer: *In Vitro* Studies

Several *in vitro* studies have been carried out to test the usefulness of *L. rhamnosus* GG in anti-cancer therapeutic practice and have focused more on the effects of this bacterial strain on metabolism, cell proliferation and invasiveness, and immunomodulation.

### Metabolism

One of the uncovered effects of the *L. rhamnosus* GG is on the polyamines metabolism. Polyamines are positively charged molecules playing a major role in DNA stabilization and cellular growth (Larqué et al., 2007); the uncontrolled cell replication leads eventually to lose the homeostatic concentration of these compounds, thus reaping cancer development. Gastric carcinoma cells (HGC-27) were exposed to LGG homogenate for 24 and 48 h. The activity of the enzymes involved in this process was observed as well as the polyamines profile; the outcome was a dose-dependent decrease of the polyamine profile, up to the 20% in the 48 h and consecutively the detection of lower cell proliferation (Linsalata et al., 2010).

### Cell Proliferation and Invasiveness

Vitamin K1 is believed to be an anti-proliferative agent; its administration in combination with viable LGG to three differently graded colon cancer cells (Caco-2, HT-29, and SW480) has shown a remarkable pro-apoptotic effect particularly on Caco-2 cells at 48 h of treatment. This achievement suggests how the combination of agents can cooperate to extend one’s functionality in reducing tumor expansion (Orlando et al., 2016). Invasiveness has been also investigated through the expression levels of matrix metalloproteinase-9 (MMP-9) and tight junction protein zona occludens-1 (ZO-1), as markers of the ECM degradation. Accordingly, metastatic colon cancer cells treated with cells free supernatants from LGG culture, achieved the gain of ZO-1 and the decrease of MMP-9 (Escamilla et al., 2012). These findings indicate an active role of the molecules released by LGG in the probiotic’s growth medium. Such molecules may reduce the infiltration property of tumor cells and the invasive and metastatic potential of cancer cells.

### Immunomodulation

The immunomodulating properties of LGG can be observed in a prospective use as a treatment, as well as a protective agent against inflammation. Neutrophils activation and dendritic cells (DC) maturation after exposure to LGG, have been explored by Cai et al. (2016), based on its suggested use against bladder cancer (Seow et al., 2010). In particular, neutrophils pre-cultured with LGG can stimulate the DCs maturation and the release of cytokines, like IL-12p70, which in turn activate the T-cells mediated immune response against the tumor environment. However, the optimal dose and time of LGG exposure is yet to be fully understood, because DCs seem to undergo exhaustion on high doses (Cai et al., 2016), thus reducing T-cells activation and the potential efficacy of the treatment. Moreover, LGG may be useful against chronic inflammation. The epithelium response to infective agents or to damages occurs also through the production of the pro-inflammatory chemokine IL-8. Of note, chronic inflammation induced by IL-8 may cause cancer development (Waugh and Wilson, 2008). LGG has proven to be effective in lowering both the *Helicobacter pylori*-induced IL-8 production and its adhesion on gastric adenocarcinoma cells (Rokka et al., 2008). Flagellin, the principal protein component of bacterial flagellum, is able to increase pro-inflammatory chemokine levels, including IL-8, through nuclear translocation of *NFκB* (Lopez et al., 2008). In particular, flagellin can cause a 17-fold increase in IL-8 production in Caco-2 cells, whereas treatments with both viable LGG and UV-inactivated LGG inhibited the degradation of the Iκ*B.* It, in turn, prevents the translocation of the transcription factor and the expression of IL-8 as an inflammatory messenger (Lopez et al., 2008). Other studies shown that peripheral blood mononuclear cells (PBMC) incubated *in vitro* with LAB, including LGG, showed a higher secretion of cytokines, proteins, or peptides acting as mediators and regulators of the immune response (Sheih et al., 2001), other results were an increase in the cytotoxic activity of natural killer cells against viruses and cancer cells (Delcenserie et al., 2008).

However, an *in vitro* study conducted on human DC and colon cancer cells (CaCo-2) treated with LGG did not highlight significant effect on inflammatory cytokine secretion. In particular, weaker non-significant effect was observed for IL-1b, IL-10, and TNFa, while no increase or decrement was observed for IL-12 secretion (Elmadfa et al., 2010).

## LGG in Cancer: *In Vivo* Animal Studies

### Protective Effects on Tumor Initiation

The first *in vivo* study showed that *L. rhamnosus* GG can interfere with the tumor promotion of dimethyl-hydrazine (DMH) induced colon cancer in 344 male Fischer rats. In particular, the effect mediated by LGG was more evident in rats receiving a high fat regimen and pre-treated with LGG (Goldin et al., 1996), supporting the idea that this microorganism can achieve a protective role against colon cancer development by inhibiting or attenuating the mutagenic effects of carcinogenic substances.

Most recently, Gamallat et al. (2016) showed that LGG induced apoptosis and reduced the expression of several angiogenetic and inflammatory proteins in rats with DMH-induced colon cancer. These data strongly support the notion that LGG administration may play a role in tumorigenesis prevention through angiogenesis and inflammation inhibition (Gamallat et al., 2016).

### Anti-inflammatory Effects during Anticancer Treatments

Besides the preventive effects of LGG on tumor development and progression, several studies in animal models showed its efficacy in preventing radiation- and chemotherapy-induced toxicities (Seow et al., 2010; Ciorba et al., 2012; Wang et al., 2017). Ciorba et al. (2012) showed that the radiation-induced epithelial damage can be prevented by administration of LGG. In this study mice were treated with LGG 3 days before the beginning of radiation therapy and observed the changes in apoptosis and crypt survival. Apoptosis levels decreased from 30 to 17% in the LGG pretreated group while crypt survival doubled. The protective effects of LGG are mediated by the activation of the Toll-like receptor-2 (TLR-2) and the shift of cycloxigenase-2 (COX-2) expression levels. Mesenchymal stem cells in the proliferative crypt region, express higher levels of COX-2 when treated with LGG (Ciorba et al., 2012). This may lead the release of elevated levels of prostaglandine-2 (PGE_2_) which, in turn, may be associated with the epithelium proliferation against radiation damage (Riehl et al., 2000; Stenson, 2008; Ciorba et al., 2012).

It is known that LGG can modulate the inflammatory state occurring during cancer development and transformation. In particular, the inflammatory reduction is one of the most important goals for cancer prevention and treatment. Seow et al. (2010) showed that LGG may induce bladder cancer regression in mice with lower inflammatory toxicity compared to common anti-cancer treatments. This study’s regimens consist in lyophilized LGG directly instilled in bladder, as well as Bacillus Calmette-Guerin (BCG) immunotherapy. Both treatments showed a considerable number of cured mice. However, in some cases the treatment with BCG showed an increased severe inflammatory response causing the delay of the scheduled treatment, whereas LGG did not cause any AEs (Seow et al., 2010), suggesting a protective role of LGG toward inflammation.

Intriguingly, Wang et al. (2017) recently described the anti-inflammatory properties of LGG. The authors showed that the protein p40, an LGG-derived protein, was able to induce class switching of B-cell to IgA-secreting plasma cells in an EGFR-dependent manner (Wang et al., 2017). It is known that IgA secretion may reduce the inflammatory response by the promotion of toxins and pathogenic antigens clearance (Mantis et al., 2011). Accordingly, the lower inflammatory response may increase the adherence to the treatments and the compliance in cancer patients.

## Clinical Evidences on LGG Administration in Cancer Patients

The most important effect of LGG, observed in cancers patients is linked to the reduction of acute or chronic diarrhea associated with chemotherapy and radiotherapy in cancer patients (Wang et al., 2016). Diarrhea affects the quality of life of patients as well as the anti-cancer treatment efficacy. Indeed, such patients experiencing diarrhea cannot tolerate the standard dose of therapy and need many adjustments in its dose and frequency. This side effect may also require hospitalization caused by the electrolytes loss. Acute or chronic diarrhea is observed among cancer patients treated with pelvic radiation therapy and/or with common antineoplastic agents. In detail, microvascular apoptosis and sclerosis of the vessels may occur during radiation therapy as results radiation-induced gut toxicity (RIGT) (Stansborough et al., 2017); while direct epithelial damage may be produced by chemotherapy (Van Sebille et al., 2015). Drugs used to stop diarrhea, such as loperamide, were not useful to restore the gut microflora. While the appropriate use of probiotics has been shown to be active and safe (Wang et al., 2016). For instance, Osterlund et al. (2007) reported interesting results in the phase III randomized study on cancer patients. The authors considered the combination of radio- and chemo-therapy and radiation therapy alone. The LGG was administered as 1–2 capsules/die (10^10^ Colony Forming Units) for 24 weeks during any anti-cancer treatment. The reports for diarrhea episode of grades 3 and 4 were 15% lower for those receiving LGG than the control group, suggesting that LGG supplementation may be a well-tolerated product able to ameliorate the compliance to the common cancer therapies (Osterlund et al., 2007).

Recently a meta-analysis of 11 randomized and placebo controlled studies evaluating the efficacy and safety of probiotics for prevention of chemo- and radiotherapy-induced diarrhea in people with abdominal and pelvic cancer was published. The study reported a potential beneficial effect of probiotics in the prevention of chemoradiotherapy-induced diarrhea with rare AEs (Wang et al., 2016).

## Safety

After decades of administration of probiotics in clinical practice, some concerns on their safety have arisen due to the exponentially increased use and availability.

To best of our knowledge, the last guideline, reporting the potential side effects of probiotics, was described by the Joint FAO/WHO Working Group in 2002 (FAO/WHO, 2002). Systemic infections, deleterious metabolic activities, excessive immune stimulation in susceptible individuals and gene transfer were hypothesized. In particular, the report recommends the assessment of probiotics health claims and their safety use in a benefit patient’s prospective (FAO/WHO, 2002).

Many scientific studies have shown the therapeutic safety of the administration of probiotics, especially LGG. However, some studies have been reported bacteremia due to LGG overexpression, although these studies take into account particular classes of individuals such as children or diabetic patients (Land et al., 2005; Zein et al., 2008).

For a better understanding of the potential and rare AEs related to the consumption of probiotics, it is crucial to focus on their strain-by-strain characteristics (Marco et al., 2006; Shanahan, 2012). Indeed, it was observed that phenotypical differences within the same species, such as *Lactobacillus rhamnosus*, and different strain might be related to the behavioral switch from symbiotic to opportunistic *L. rhamnosus*, isolated from bacteremia, compared to the commercial *Lactobacillus* GG (ATCC 53103) (Ouwehand et al., 2004). However, such switch from symbiotic to opportunistic species is not yet assessed. Therefore, as reported below, a deep study of clinical records and/or case reports published in the literature remains the best approach to finding out any adverse effects of probiotics, such as LGG.

### LGG Administration in Healthy Subjects

In a 5-year follow-up study, healthy-term infants, previously chosen for a double-blind controlled multicenter study, received LGG during the 1st year of age (Scalabrin et al., 2009). No serious AE correlated to the early consumption of LGG were described at the follow-up (Scalabrin et al., 2017). Intriguingly, it was shown that perinatal administration of LGG reduced allergy tendency in infants (Lundelin et al., 2017). Salminen et al. (2002) analyzed the effects of LGG consumption and lactobacilli bacteremia within 11-years period (1990–2000) in a large population. The study showed that only 11 cases were attributable to LGG and no time-related incidence was reported, deducing that no correlation between probiotic use of LGG and LGG bacteremia was observed (Salminen et al., 2002). Finally, no AEs after LGG consumption were observed among healthy elderly people, as reported in the study by Hibberd et al. (2014).

### LGG Administration in Cancer Patients

To date, regarding the use of LGG in the oncology practice, there are no studies showing the onset of complications due to the administration of probiotics. With particular reference to oncologic pathologies, only two cases of bacteremia have been reported following the administration of probiotics. Both patients had onco-hematologic tumors and in both cases the development of bacteremia was attributable not to tumor but to bone marrow transplantation and to immunosuppressive therapies performed by patients (Majcher-Peszynska et al., 1999; Robin et al., 2010). These data show that the use of LGG in support of cancer therapy has a high level of safety, although very rarely were recorded episodes of bacteremia.

Redman et al. (2014) very nicely summarized the safety of probiotics combined with anti-cancer therapies. The authors analyzed 17 studies, including 1530 people (756 probiotics administered, 774 not consuming probiotics). The overlook for safety has been conducted considering different probiotics such as *Saccharomyces boulardii*, several species of *Lactobacillus* including LGG and *Bifidobacterium* together with *Streptococcus thermophilus* and VLS#3 (a multispecies formula). Only rare AEs relate to probiotics administration were described but none of them was associated with the use of LGG even during neutropenia caused by chemotherapy (Redman et al., 2014).

## Conclusion

Decades of clinical practice have experienced the use of probiotics as food supplies that can help patients, most likely affected by intestinal dysbiosis or by concomitant diseases. Since the time it was isolated, LGG has proven to be effective, through *in vitro* and *in vivo* experiments against inflammation, epithelial damage, invasiveness and proliferation of malignancies. Several studies on humans have been conducted to assess its usefulness against AE such diarrhea experienced by cancer patients. Even though the lack of studies homogeneity, the administration of LGG is clearly safe.

Well-designed clinical trials are mandatory to define the appropriate use of LGG during anti-cancer treatments, including pelvic radiation therapy and chemotherapy. According to the results obtained by such clinical trials, LGG may be regulated based on their use. We are confident that LGG may exert an important role in preserving the gut microbiota during such treatments and in improving the quality of life. Such positive effects of LGG administration may also enhance the adherence and compliance to treatments.

## Author Contributions

GB, FT, FM, and FF critically contributed to elucidate the clinical aspects of the probiotics use in cancer therapy. MS contributed to discuss the characterization of LGG. RS, EC, LF, and ML discuss the role of LGG in pre-clinical studies. The manuscript was amended based on comments from all authors. All authors read and approved the final version of the manuscript.

## Conflict of Interest Statement

ML is the PI of a research grant founded by Dicofarm Spa to his University Department. The other authors declare that the research was conducted in the absence of any commercial or financial relationships that could be construed as a potential conflict of interest.
